# Ultrasound and Microbubbles for Targeted Drug Delivery to the Lung Endothelium in ARDS: Cellular Mechanisms and Therapeutic Opportunities

**DOI:** 10.3390/biomedicines9070803

**Published:** 2021-07-12

**Authors:** Rajiv Sanwal, Kushal Joshi, Mihails Ditmans, Scott S. H. Tsai, Warren L. Lee

**Affiliations:** 1 Keenan Research Centre for Biomedical Science, St. Michael’s Hospital, Unity Health Toronto, Toronto, ON M5B 1T8, Canada; rajiv.sanwal@mail.utoronto.ca (R.S.); kushal.joshi@ryerson.ca (K.J.); misha@ditman.ca (M.D.); scott.tsai@ryerson.ca (S.S.H.T.); 2 Department of Laboratory Medicine and Pathobiology, University of Toronto, Toronto, ON M5S 1A8, Canada; 3 Department of Mechanical and Industrial Engineering, Ryerson University, Toronto, ON M5B 2K3, Canada; 4 Institute of Biomedical Engineering, Science and Technology (iBEST), Toronto, ON M5B 1T8, Canada; 5 Biomedical Engineering Graduate Program, Ryerson University, Toronto, ON M5B 2K3, Canada; 6 Interdepartmental Division of Critical Care Medicine, University of Toronto, Toronto, ON M5S 1A1, Canada

**Keywords:** acute respiratory distress syndrome, ultrasound, microbubbles, endothelial cells, vascular leak, drug and gene delivery

## Abstract

Acute respiratory distress syndrome (ARDS) is characterized by increased permeability of the alveolar–capillary membrane, a thin barrier composed of adjacent monolayers of alveolar epithelial and lung microvascular endothelial cells. This results in pulmonary edema and severe hypoxemia and is a common cause of death after both viral (e.g., SARS-CoV-2) and bacterial pneumonia. The involvement of the lung in ARDS is notoriously heterogeneous, with consolidated and edematous lung abutting aerated, less injured regions. This makes treatment difficult, as most therapeutic approaches preferentially affect the normal lung regions or are distributed indiscriminately to other organs. In this review, we describe the use of thoracic ultrasound and microbubbles (USMB) to deliver therapeutic cargo (drugs, genes) preferentially to severely injured areas of the lung and in particular to the lung endothelium. While USMB has been explored in other organs, it has been under-appreciated in the treatment of lung injury since ultrasound energy is scattered by air. However, this limitation can be harnessed to direct therapy specifically to severely injured lungs. We explore the cellular mechanisms governing USMB and describe various permutations of cargo administration. Lastly, we discuss both the challenges and potential opportunities presented by USMB in the lung as a tool for both therapy and research.

## 1. Introduction

Endothelial cells line the entire vascular system and are the interface between blood and tissue [1]. A primary role of the microvascular endothelium is to regulate the flux of molecules between the vascular lumen and the surrounding tissue parenchyma. A characteristic feature of the endothelium is heterogeneity between different-sized vessels (e.g., macrovascular, such as the aorta, vs. microvascular in capillaries) and between different tissues. For instance in the lung, microvascular endothelial cell permeability is relatively low and tightly regulated by cell junctional complexes, of which major protein components include vascular endothelial (VE)-cadherin, claudin-5 and occludins. By comparison, the endothelial cells in the liver sinusoids have intercellular gaps that permit the free movement of fluid and circulating components in and out of the vascular lumen.

The role of endothelial dysfunction and increased permeability in the pathogenesis of inflammatory disease has been appreciated for decades [2], but interest in its potential modulation as a therapy has grown with the COVID-19 pandemic; there has also been a broader realization of the importance of endothelial leakage in sepsis and lung injury [3,4]. Therapies that decrease endothelial permeability would be of particular interest in the lung, where acute respiratory distress syndrome (ARDS, also called acute lung injury) is characterized by disrupted alveolar endothelial and epithelial barriers, leading to pulmonary edema and arterial hypoxemia [5]. It occurs in nearly 10% of all intensive care unit (ICU) admissions and results in a mortality rate of up to 40% despite the best supportive care [6]. ARDS is usually caused by an excessive host inflammatory response to an infectious (e.g., pneumonia, sepsis) or non-infectious insult (e.g., trauma), resulting in endothelial and/or epithelial damage and fluid extravasation [7].

Treatment of ARDS is difficult due to an incomplete understanding of its pathophysiology but also because of an inability to target severely injured regions of the lung. In this article, we first briefly review some fundamental determinants of endothelial activation and permeability; subsequently, we focus our discussion on how the combination of thoracic ultrasound and intravenously administered microbubbles (USMB) may permit preferential delivery of therapeutic cargoes to the lung endothelium in the most damaged regions of the lung [3,8].

### 1.1. Endothelial Activation and Leakage during Inflammation

#### 1.1.1. Pulmonary Endothelial Inflammation and Vascular Permeability 

Endothelial cells undergo phenotypic changes after the binding of pro-inflammatory cytokines such as interleukin-1β (IL-1β) and tumor necrosis factor-α (TNF-α) to their receptors [9]. The most notable changes are an upregulation of cellular adhesion molecules such as intercellular adhesion molecules (ICAM), vascular cell adhesion molecules (VCAM), and endothelial cell-leukocyte adhesion molecules (ELAM), which facilitate the binding of leukocytes [10,11]. ICAM-1 has been well documented to be increased in the serum and lung tissue of ARDS patients [12,13]. Its knockout also showed an attenuation of edema in a septic mouse model attributed to impaired leukocyte recruitment to the site of injury [14]. The levels of E-selectin, the endothelial selectin, are also elevated in ARDS patients [15]. Despite their involvement in the disease, however, therapies targeting the selectins have yielded mixed results. Baboons with sepsis-induced ARDS did not respond to anti-E-selectin and L-selectin antibody therapy, despite an antibody targeting both selectins being protective in pigs [16,17].

Endothelial cells also contribute to inflammation by secreting pro-inflammatory cytokines themselves, directing immune cells to the site of injury and leading to an increase in vascular leakage [18,19]. Increased cytokines have been found in the alveolar lavage fluid of ARDS patients [20,21] and, while necessary for the immune response, may contribute to tissue injury. Cytokines such as IL-6 can induce endothelial permeability [22]; others, such as IL-1β and TNF-α, are potent chemoattractants for leukocytes. The sensing of endogenous or exogenous inflammatory signals (e.g., bacterial lipopolysaccharide, viral nucleic acid) triggers the formation and activation of the inflammasome in both immune cells and lung parenchymal tissue. The inflammasome is a multiprotein signaling complex that catalyzes cleavage and release of IL-1β and IL-18 and has been shown to contribute to ARDS (as reviewed in [23]); inflammasomes consist of sensor, adaptor and effector components such as the NOD-like receptor, pyrin domain containing 3 (NLRP3) protein which detects intracellular stress (as reviewed in [24]). Activation of the NLRP3-inflammasome has been postulated to contribute to lung injury in COVID-19, and blockade of pro-inflammatory cytokines with antibodies is a promising therapeutic strategy [25,26,27,28,29].

Excessive inflammation, as seen in ARDS, causes barrier disruption and fluid leakage into the interstitial space. This is mediated through various mechanisms including the previously mentioned cytokines, an increase in reactive oxygen species production by activated leukocytes and the production of neutrophil extracellular traps (NETs) (e.g., in patients suffering from critical illness due to COVID-19) [30,31,32]. 

**Figure 1:**
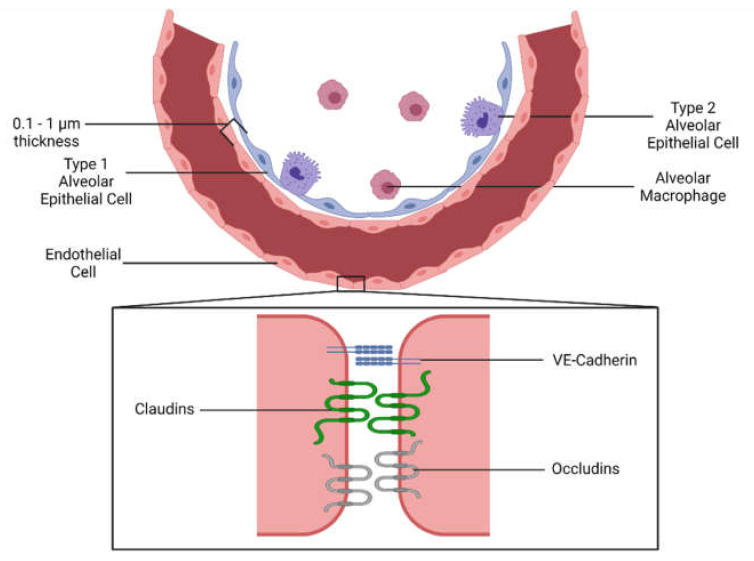
Anatomy of the alveolus. The alveolus is the basic functional unit of the lung. An extremely thin alveolar wall allows for gas exchange between air and blood. The outer layer is composed of pulmonary epithelial cells (also known as pneumocytes), of which there are two types. Type I epithelial cells permit gas exchange, while type II epithelial cells produce pulmonary surfactant. Pulmonary endothelial cells form the inner layer of the wall and line the pulmonary microvasculature. Endothelial cells form a continuous layer formed through tight and adherens cellular junctions between neighboring cells, thus preventing fluid leakage out of the lung. Vascular endothelial cadherin (VE-cadherin), claudins, and occludins form these junctions through homophilic and/or heterophilic interactions, restricting junction width to 2–5 nm. Created with BioRender.com.

Much of the barrier integrity in the continuous microvascular endothelium is conferred by the adherens junctions, in which homophilic interactions between VE-cadherin hold adjacent cells together tightly [33,34] (Figure 1). A decrease in cell-surface VE-cadherin is an important step for the extravasation of leukocytes through selective tyrosine phosphorylation and internalization [11,35,36]. In the context of lung injury, a decrease in VE-cadherin is mediated by multiple signals, including TNF-α, thrombin, and vascular endothelial growth factor (VEGF) [34]. Other non-inflammatory signaling molecules can decrease permeability. Angiopoietin-2 is a well-characterized negative regulatory ligand for receptor tyrosine kinase Tie2, inhibiting its phosphorylation and increasing endothelial permeability [37]. Levels of angiopoietin-2 act as a marker of clinical outcome and injury severity in trauma and sepsis patients [38,39]. The angiogenic cytokine VEGF is also higher in critically ill patients; paradoxically, while higher VEGF levels are associated with a poor prognosis, it is not predictive of lung edema [39,40]. 

More recently, additional players have been identified as being involved in the disrupted lung endothelium in ARDS [41]. The forkhead box protein M1, an important transcription factor involved in cell proliferation, is key in repairing the pulmonary endothelium in multiple models of murine lung injury [42,43]. Various microRNAs (miRs) are also of interest due to their crucial roles in the regulation of the expression of multiple genes: miR-150 levels are inversely correlated with the severity of disease in ARDS patients, and it has been demonstrated to decrease the severity of lung injury by repairing cellular junctions [44,45]. miR-26a-5p, through the reduction in connective tissue growth factor, protected mice with lung injury from lipopolysaccharide (LPS, i.e., endotoxin) by decreasing the severity of inflammation [46]. Finally, fibroblast growth factor 2 and phospholipase D2 have been reported to stabilize VE-cadherin—the former by inhibiting pro-inflammatory pathways, and the latter by preventing VE-cadherin phosphorylation—thereby improving pulmonary endothelial barrier permeability [47,48]. 

Despite this accruing knowledge as to the regulation of lung microvascular endothelial permeability, there has been relatively little progress in its therapeutic manipulation.

#### 1.1.2. Pulmonary Endothelial Dysfunction—Coagulation 

While endothelial cells normally inhibit coagulation, when activated they initiate the coagulation cascade by the expression of tissue factor. Ultimately, the cascade produces thrombin and fibrin. Tissue factor-induced coagulation is a significant contributor to mortality in critical illness and play a large role in ARDS [49,50]. In addition to being a direct pro-coagulant, thrombin interferes with the endothelial barrier and exacerbates vascular leakage through binding to its receptor proteinase-activated receptor 1 (PAR1) [51]. Activated endothelial cells also exhibit an inhibition of their normally pro-fibrinolytic state due to an upregulation of the plasminogen activator inhibitor-1 (PAI-1) [52]. Indeed, endothelial cells collected from the lungs of ARDS patients demonstrate an increase in both thrombin and PAI-1 [53]. Of note, microvascular thrombosis has been recognized as playing a role in the pathology of patients with severe COVID-19 [54]. A study comparing ARDS lung autopsies between COVID-19 patients and influenza patients found a nine-fold higher incidence of microvascular thrombosis in the lungs of COVID-19 patients [55]. 

### 1.2. Current Therapies Targeting the Endothelium 

Given the central role of the lung microvascular endothelium in the pathogenesis of ARDS and sepsis, numerous approaches to modulate endothelial cell activation and decrease vascular leakage are being investigated [56]. For example, the sphingolipid sphingosine-1-phosphate (S1P) is a potent enhancer of the endothelial barrier while also acting to regulate systemic cytokine levels [22,57]. Very recently, Akhter et al. used an endothelial-specific S1P receptor-1 (S1PR1) knockout animal model to investigate the role of S1P in pulmonary endothelial regeneration in an endotoxemia injury model [58]. They found that S1P1R deletion resulted in increased endothelial permeability. Furthermore, they determined that S1PR1-expressing endothelial cells are required for barrier repair, and that these cells were reprogrammed to increase the production of S1P, suggesting therapeutic potential for the S1P pathway in endothelial injury.

There has also been extensive interest in targeting the angiopoietin receptor Tie2 and its signaling axis as a means to restore the endothelial barrier. For example, the Tie2 agonist vasculotide was shown to decrease vascular leakage, tissue edema and organ dysfunction in multiple animal models of sepsis or lung injury [59,60,61]. The simultaneous sequestration of the inhibitory ligand angiopoietin-2 and the activation of Tie2 was shown to restore endothelial barrier integrity and reduce mortality in septic animals [62]. Targeting the Tie2 pathway combined with decreasing circulating VEGF rescued septic mice by reducing ICAM-1 levels, decreasing inflammation, and improving endothelial barrier strength [63]. Increasing the activity of Tie2 by means of the inhibition of VE-protein tyrosine phosphatase, a Tie2-inactivating protein, also enhanced the endothelial barrier in the lungs and retinas of mice [64,65]. 

More generally, it is important to point out that a number of groups have now independently shown that decreasing vascular leakage can be accomplished without impairing innate immunity, highlighting the fact that tissue edema and leukocyte recruitment can be controlled separately [2].

Despite these conceptual and therapeutic advances, targeting the endothelium for therapy remains challenging, especially in the injured lung. Nanoparticle-enhanced delivery systems are the subject of intense research [66]. Inhalation of nanoparticles did show transit into the vascular space, suggesting that the endothelial barrier is reached [67]. The specific composition of nanoparticles can regulate their delivery, as demonstrated by one group showing the specificity of their nanoparticles for the alveolar endothelium, with or without cargo [68,69,70]. To increase endothelial specificity even further, antibodies and ligands for endothelial-specific receptors have been added to the nanoparticles. For example, peptides against sialic acid, ephrin type-A receptor 2, and ICAM have all been described [71,72,73,74].

Nonetheless, a major issue is how to preferentially deliver therapeutic cargo to the most injured regions of the lung in ARDS. This is particularly problematic given the heterogeneity of the lung in ARDS, in which severely consolidated areas in the lungs of a given patient may be abutting relatively aerated regions [75,76]. Intravenous administration of drugs (including nanoparticles) may result in undesirable off-target effects in other organs such as the kidney or liver. Hypoxic vasoconstriction (the normal narrowing of vessels in the lung in response to hypoxia) may complicate this further by decreasing the amount of drug able to reach the tissue. Inhaled agents are preferentially distributed to the best ventilated areas of the lung, potentially neglecting the most injured regions; furthermore, they make first contact with the lung epithelium, potentially decreasing access to the lung endothelium. Thus, there is a need for a delivery method which can achieve a high rate of lung endothelial delivery and preferentially targets the most injured lung areas.

## 2. Introduction to Ultrasound-Microbubble Mediated Therapy

**Figure 2:**
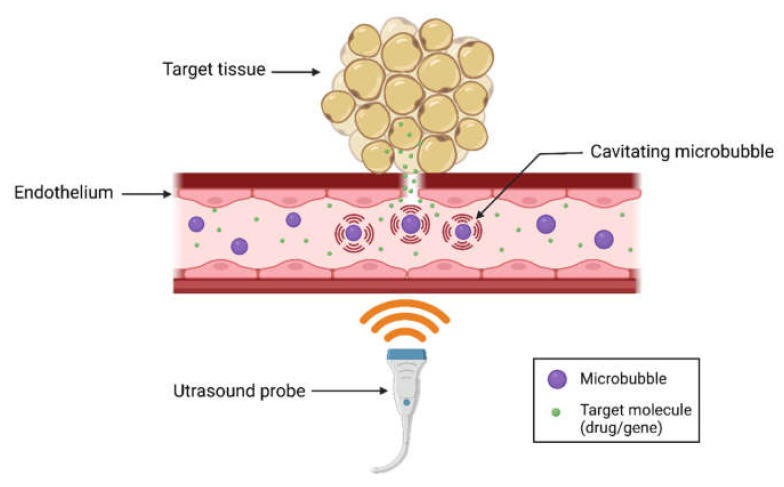
Basic principle of ultrasound-microbubble (USMB)-mediated drug and gene delivery. When microbubbles flowing through the blood are exposed to the ultrasound field, they undergo rapid changes in size and shape (i.e., cavitation), inducing high shear stresses on the surrounding endothelium. This increases vascular permeability and cellular internalization (e.g., endocytosis), leading to targeted delivery of drugs/genes in the area of insonation. The result is enhanced delivery of therapeutic cargo to the local endothelium itself and to the organ being perfused. Created with BioRender.com.

Over the past two decades, ultrasound-microbubble (USMB)-mediated intracellular drug and gene delivery has emerged as a promising therapeutic approach for the treatment of the endothelium. One of the first reports on USMB-mediated intracellular delivery appeared in the late 1990s when ultrasound and commercial ultrasound imaging contrast microbubbles (Albunex^®^) were used for the transfection of cultured Chinese hamster ovary cells with a pGL2 luciferase reporter plasmid [77]. Since then, USMB-mediated delivery has been used for a variety of in vitro and in vivo applications such as delivering drugs across the blood–brain barrier, transfecting liver cancer cells with genes for therapy as well as delivering drugs to endothelial cells [78,79,80,81]. The principle of the method is as follows: 

When circulating microbubbles are exposed to ultrasound, they grow and shrink in response to the alternating low- and high-pressure portions of the acoustic wave (i.e., cavitation). These oscillations of microbubbles exert mechanical forces on the endothelium, which increases local vascular permeability and facilitates delivery of external molecules into targeted tissues [82] (Figure 2). Depending on the intrinsic properties of the microbubbles and applied ultrasound parameters, microbubbles may undergo stable oscillations (i.e., stable cavitation) or violent oscillations and collapse (i.e., inertial cavitation) [83]. Microbubble oscillations induce acoustic microstreaming flows in the surrounding medium while violent collapse leads to the formation of high-velocity microjets and shockwaves which induce shear stresses on the endothelium, leading to intracellular and trans-endothelial delivery of drugs or plasmids [84,85]. Since microbubbles undergo cavitation only in the presence of an ultrasound field, the delivery is highly targeted towards the specific area of insonation, thus reducing off-target effects. 

Microbubbles used for this application often contain a high molecular weight perfluorocarbon gas core surrounded by a protective shell made from lipids, proteins or polymers which improve their stability and lifetime [86]. The microbubbles can be co-administered intravenously along with drugs or other cargo, or can be designed to carry the therapeutic cargo inside the bubble or bound to the microbubble surface [87].

Due to its ease of application, non-invasive and highly localized nature, USMB-mediated drug/gene delivery is an attractive option for the treatment of the endothelium. The endothelium is known to play a crucial role in several vascular pathologies including arterial restenosis, arteriosclerosis, thrombosis, and hypertension [88]. To date, several research groups have demonstrated the utility of USMB-mediated delivery for endothelial treatment. For example, USMB has been utilized for successful intra-endothelial delivery of plasmid DNA encoding a phosphomimetic variant of the endothelial nitric oxide synthase (eNOS S1177D) gene, enhancing the production of nitric oxide (NO), which is a crucial mediator of anti-atherosclerotic effects [89]. Another study utilized USMB-mediated delivery of rapamycin (an antiproliferative drug) for successful inhibition of neointima formation, a condition resulting from endothelial denudation, in rat carotid arteries [90]. 

The cellular mechanisms of how USMB enhances drug delivery include sonoporation and endocytosis; these will be briefly reviewed here. 

### 2.1. Mechanisms of USMB-Mediated Intracellular Delivery

#### 2.1.1. Sonoporation

The term ‘sonoporation’ refers to the formation of transient pores in the cell membrane upon exposure to ultrasound-activated microbubbles. The formation of pores enhances membrane permeability, thus facilitating intracellular delivery of external molecules. Several investigations have been carried out to characterize these membrane pores. Using indirect molecular probing and direct visualization using scanning-electron microscopy (SEM), one study revealed the formation of membrane pores in the size range of 75–100 nm following USMB treatment of MAT B III cells [91]. Another study utilized transmembrane current measurements of *Xenopus laevis* oocytes for real-time monitoring of sonoporation, following treatment with ultrasound and commercial Definity^®^ microbubbles. The results indicate the formation of membrane pores of radius 110 ± 40 nm [92]. Several other investigations have been performed to characterize the membrane pore size following USMB treatment using advanced atomic force microscopy and electron microscopy techniques [93,94,95,96,97]. 

The membrane pores formed by sonoporation are transient and reseal within a few seconds or minutes. Few researchers have investigated the temporal dynamics of membrane resealing following sonoporation by cavitating microbubbles. Using live-cell fluorescence microscopy and the fluorescent dyes Fura-2 and propidium iodide, one study observed membrane resealing in endothelial cells within 5 s of exposure to single-shot pulsed ultrasound with microbubbles [98]. Another recent investigation utilized real-time confocal fluorescence microscopy to visualize membrane resealing and observed that the resealing process could be completed with 1 min, depending on the initial pore size [99]. All these studies have provided strong visual evidence of membrane perforation and resealing following USMB treatment, suggesting sonoporation as a major mechanism of USMB-mediated cargo delivery. 

Why USMB induces sonoporation has been explained by physical phenomena such as acoustic microstreaming, shock waves and microjets generated by the cavitating microbubbles in the surrounding medium; these will be briefly discussed in the next section. 

##### Acoustic Microstreaming 

Ultrasound-driven, oscillating microbubbles undergoing stable cavitation are known to generate steady vortical flows in the surrounding liquid (i.e., swirling motion of surrounding liquid), frequently referred as acoustic microstreaming [100]. It is suggested that these microstreaming flows induce a shear stress on the nearby cells resulting in tension and stretching of the membrane, thereby inducing transient membrane perforation [101,102]. One of the earliest experimental studies on microstreaming-induced sonoporation was performed with an ultrasonic horn transducer (also known as Mason horn, sonotrode, ultrasonic homogenizer or disintegrator) vibrating at 21.4 kHz inside a Jurkat lymphocyte suspension [103,104]. These experiments suggested that microstreaming flows generated by the vibrating Mason horn induce a shear stress of 12 Pa, which is sufficient to induce sonoporation in Jurkat lymphocytes upon exposure for 7 min. Theoretical calculations performed by the same research group indicated that acoustic microstreaming flows generated by microbubbles could induce shear stresses of similar or higher magnitude, thus facilitating sonoporation [101]. Using a combination of particle image velocimetry (PIV) and theoretical analysis, other researchers have shown that the shear stresses induced by acoustic microstreaming flows around microbubbles are significantly higher (~19 Pa) than the shear stresses induced by normal blood flow (~0.5–2 Pa), resulting in sonoporation [102]. These and several other theoretical and experimental studies showed that acoustic microstreaming may be a major mechanism of sonoporation [105,106,107]. Furthermore, microstreaming flow pattern is strongly dependent on the driving ultrasound frequency, microbubble size, pressure amplitude, properties of surrounding media as well as oscillation mode of microbubbles, which might explain why sonoporation and intracellular delivery of molecules are strongly affected by these parameters, as noted in several other studies [102,108,109,110]. 

##### Shock Waves and Liquid Microjets 

Besides acoustic microstreaming, shockwaves and liquid microjets erupting from a collapsing microbubble undergoing inertial cavitation are suggested as possible mechanisms of sonoporation. Cavitating microbubbles undergoing symmetrical bubble collapse generate a strong shock wave in the surrounding medium which may exert a significant shear stress to perforate the membrane. Some experimental studies show that the amplitude of a shock wave generated by a single collapsing bubble can be as high as 1 GPa; however, this shock dies down rapidly and can only make an impact at distances comparable to the initial microbubble radius [100]. However, in the presence of multiple microbubbles, shockwaves emitted by several collapsing bubbles may combine and make an impact over larger distances [100]. Some molecular dynamics simulation studies have shown that a lipid bilayer membrane exposed to shockwaves may undergo compression and rebound, resulting in the formation of membrane pores during the reorganization of the lipid bilayer [111,112,113]. Though this study did not consider shockwaves erupting from collapsing bubbles, the mechanism may be similar for sonoporation induced by collapsing bubble shockwaves.

Fundamental fluid mechanics studies have revealed that a cavitating microbubble near a wall or a solid boundary collapses asymmetrically, ejecting fast liquid microjets towards the wall [114,115]. It has been hypothesized that when a microbubble undergoes inertial cavitation near the walls of blood vessels, the microjets ejected from asymmetrical collapse exert high shear stresses on the endothelial cells, causing membrane poration [116]. Some experimental studies conducted in the late 2000s have supported this hypothesis [94,98,117]. 

#### 2.1.2. Endocytosis

For several years, sonoporation was thought to be the major mechanism of USMB-mediated intracellular delivery. However, mounting evidence now suggests that endocytosis may also play a major role. Experiments with primary endothelial cells exposed to USMB in the presence of fluorescent dextrans (4.4 to 500 kDa) have shown localization of 155 and 500 kDa dextrans in distinct vesicles and uniform distribution of 4.4 and 70 kDa dextrans throughout the cytosol [118]. Furthermore, this study showed a significant decrease in the intracellular delivery of all dextran molecules after independent inhibition of caveolin-mediated endocytosis, clathrin-mediated endocytosis and micropinocytosis pathways. Additionally, adenosine triphosphate (ATP) depletion showed a reduced uptake of 4.4 kDa dextran with no uptake of 500 kDa after USMB treatment, consistent with the inhibition of endocytosis (which requires ATP) rather than an effect on sonoporation. Using 3D fluorescence microscopy, this study also demonstrated colocalization of 500 kDa dextran vesicles with caveolin-1 and clathrin [118]. Together, this evidence suggests that endocytosis is a major mechanism of USMB-mediated intracellular delivery, especially for larger molecules. 

In another study, C6 rat glioma cells were treated with either chlorpromazine (a non-specific inhibitor of clathrin-mediated endocytosis) or genistein (used to inhibit caveolae-mediated endocytosis) before exposure to USMB in the presence of the fluorescent dye SYTOX green [119]. The results showed a 2.5-fold increase in the SYTOX uptake time constant for the chlorpromazine-treated group, and a 1.1-fold increase in the uptake time constant for the genistein-treated group, indicating the dominance of the clathrin-mediated pathway in USMB-mediated endocytosis. Unfortunately, the lack of specificity of the inhibitors limits the interpretation of this study [119]. Similar results were obtained in another investigation which showed increased clathrin content per clathrin-coated pit and enhanced clathrin-mediated endocytosis within 5 min of USMB exposure [120]. Some researchers have also studied the influence of ultrasound parameters on the uptake mechanisms (i.e., sonoporation vs. endocytosis). When human melanoma cells were subjected to USMB in the presence of fluorescein isothiocyanate (FITC)-dextran at different acoustic pressures (100–500 kPa), endocytosis, as evidenced by the presence of dextrans in vesicles, appeared to be a dominant mechanism of cellular uptake at low acoustic pressures. In contrast, sonoporation appeared to be more dominant at higher acoustic pressures [121]. The dependency of uptake mechanisms on applied acoustic pressures may be linked to the acoustic behavior of microbubbles, which change at different acoustic pressures. 

##### Biological Mechanisms of USMB-Induced Endocytosis 

Several biological mechanisms of USMB-induced endocytosis have been suggested in the literature [122]. Some studies have shown that when cells are exposed to USMB or just ultrasound, a strong influx of Ca^2+^ ions is induced [118,123,124]. It is believed that the high intracellular Ca^2+^ concentration stimulates endocytosis; however, the exact mechanism is unclear [122]. It has also been suggested that the increase in reactive oxygen species (ROS) production upon exposure to ultrasound stimulates endocytosis by a ROS-dependent mechanism such as caveolae internalization due to ROS-induced caveolin-1 phosphorylation or by increasing the calcium influx [122,123,125]. However, the detailed mechanism of ROS-activated endocytosis remains elusive. According to another hypothesis, the exocytosis of lysosomes plays a crucial role in the regulation of clathrin-dependent endocytosis induced by USMB treatment [122]. The evidence for this comes from the observation that the lysosomal marker protein (Lamp-1) accumulates in the plasma membrane at the same time as USMB-induced endocytosis occurs, while the inhibition of lysosome exocytosis significantly reduces the activity of the transferrin receptor, which plays a crucial role in clathrin-dependent endocytosis [120,122]. At present, however, the precise biological mechanisms of USMB-induced endocytosis are not fully understood and more study is necessary.

##### Physical Mechanisms of USMB-Induced Endocytosis 

Endocytosis is thought to be initiated by direct physical interactions between microbubbles and the cells or through certain biological pathways triggered by these physical interactions [122]. Though the exact physical mechanisms responsible for triggering endocytosis remain elusive, certain hypotheses have been proposed. As discussed earlier, oscillating or collapsing microbubbles produce microstreaming flows, shockwaves or liquid microjets in the surrounding liquid. The shear stresses produced by these physical phenomena may induce membrane deformations and trigger endocytosis, as noted in some earlier investigations [126,127]. A second hypothesis states that the forces exerted on the plasma membrane due to microbubble–cell interactions can be transmitted downstream to the cytoskeleton, leading to cytoskeleton remodeling [122]. This process may in turn activate mechano-sensors such as integrins or stretch-activated ion channels, triggering certain endocytotic pathways [122]. Another hypothesis suggests that endocytosis is not an independent phenomenon but is triggered by sonoporation caused by exposure to USMB [122]. According to this hypothesis, formation of lysosomal patches and exocytosis is essential for sealing large membrane pores caused by USMB treatment. The lysosomal acid sphingomyelinase released during the exocytosis process converts sphingomyelin in the plasma membrane to ceramide, leading to inward budding and formation of vesicles, which is considered to be a prelude to the endocytosis process [122]. Though multiple hypotheses exist, there is no consensus on the physical mechanisms triggering USMB-induced endocytosis and more investigations need to be performed in this area. 

In summary, sonoporation and endocytosis both lead to intracellular delivery of molecules; however, it is difficult to say which one of the two dominates. The size of the molecules and the acoustic parameters are likely critical determinants [118]. Endocytosis may also be an unavoidable consequence of sonoporation and may be required for resealing of membrane pores [122]. Relative to sonoporation, endocytosis is an active and regulated pathway for the delivery of molecules and is therefore considered by some to be potentially safer for cargo delivery [122]. 

#### 2.1.3. Cargo Delivery Using USMB—On, Inside, or Around?

Different approaches for delivering cargo using microbubbles have been described. Here, we will discuss the three most common approaches used for various USMB applications, namely—binding the cargo to the microbubble shell using electrostatic or covalent interactions, embedding cargo inside the microbubble shell and co-administration of cargo and microbubbles. Though the approaches discussed here are not specific to the treatment of endothelium, the same strategies could be useful for treating lung endothelial dysfunction using USMB. 

##### Electrostatic or Covalent Binding of Cargo to the Microbubble Shell 

Several attempts have been made to bind cargo (i.e., drugs or plasmids) to the microbubble shell using electrostatic or covalent interactions [128]. Due to the proximity of the cargo to the microbubble shell, it is speculated that this approach leads to enhanced delivery efficacy due to cavitation effects [129]. In addition, the release of cargo could be limited to the area of insonation, thus reducing any adverse side-effects related to free circulating drug [129]. Plasmids, which have an inherent negative charge, can be bound onto the shells of cationic lipid microbubbles using electrostatic interactions. This approach ensures that the plasmid DNA is protected from degradation by endonucleases, thereby increasing local DNA concentration for USMB-mediated transfection [130]. Cationic microbubbles bound with plasmid DNA have been used to transfect click beetle luciferase (CBLuc) plasmids into endothelial cells using USMB treatment in vitro [130]. In another investigation, researchers used cationic microbubbles loaded with short hairpin RNA interference therapy targeting prolyl hydroxylase-2 (shPHD2) plasmid for USMB-assisted myocardial transfection to protect the heart from myocardial infarction [131]. Cationic microbubbles are usually made by incorporating cationic lipids such as 1,2-dioctadecanoyl-3-trimethylammonium-propane (DSTAP) into the shells of lipid microbubbles or by coating the shell with cationic polymers such as poly-(allylamine hydrochloride) when using albumin microbubbles [130,132]. The plasmid loading capacity is limited by the surface area of microbubbles. The maximum plasmid loading capacity usually ranges from 0.001 to 0.005 pg/μm^2^ [133]. In order to increase the plasmid loading capacity, a layer-by-layer construction of microbubbles with alternate plasmid (anionic) and poly-L-lysine (cationic) layers has been proposed [133]. Using this technique, the plasmid loading capacity has been reported to increase over 10-fold using five paired layers [133]. 

Apart from plasmids, various other cargos such as drug-loaded liposomes or nanoparticles have been bound to the surface of microbubbles. For example, doxorubicin (DOX)-loaded liposomes were covalently attached to lipid-shelled microbubbles via thiol-maleimide linkages and then used for USMB-mediated delivery to human glioblastoma cells [134]. The results indicated a four-fold decrease in cell viability with DOX-liposome loaded microbubbles compared to free DOX-liposomes or DOX alone [134]. 

##### Embedding Cargo Inside the Microbubble Shell 

Similar to electrostatic or covalent binding, embedding the cargo inside the microbubble shell could have a protective effect on the cargo, preventing its early degradation and prolonging its half-life inside the body [129]. Different types of cargo have been embedded inside the microbubble shell. In one study, rapamycin, an anti-proliferative drug, was embedded inside lipidshells of microbubbles for USMB-mediated attenuation of smooth muscle cell proliferation [90]. In vitro assays indicated that the delivery efficacy of rapamycin-loaded microbubbles was significantly higher than microbubbles co-administered with free rapamycin [90]. In another investigation, plasmid DNA encoding for the LacZ gene was incorporated into albumin microbubble shell and then used for USMB-mediated vascular gene transfection [89]. It has been demonstrated that the in vitro efficiency of transfection using plasmid-DNA embedded microbubbles is significantly higher than the transfection efficiency of the co-administration of microbubbles and plasmid [135]. 

Though both the approaches (namely binding the cargo to the microbubble shell (Section 2.1.3) and embedding the cargo inside the microbubble shell) prevent early degradation of cargo inside the body, there is no study which directly compares the cargo delivery efficacy of these two approaches. Therefore, it is difficult to say which of these two approaches is better. 

##### Co-Administration of Cargo and Microbubbles 

Co-administration of cargo with microbubbles is one of the simplest approaches for cargo delivery using USMB. In the method, a mixture of free drugs/plasmids and microbubbles is injected into the circulation or directly at the site of insonation. For example, in a recent investigation, a mixture of lipid-coated microbubbles and propidium iodide (PI) was utilized for USMB-mediated delivery of PI into endothelial cells cultured in microfluidic channels [136]. In another study, mixtures of microbubbles and evans blue dye/fluorescent-dextran were used to study USMB-mediated drug delivery to the brain in mice [137]. A mixture of commercial SonoVue microbubbles and enhanced green fluorescent protein plasmid (pEGFP)were used to study USMB-assisted gene transfection in prostate cancer cells in vitro and in vivo in a recent investigation [138]. 

#### 2.1.4. Modification of the Microbubble to Enhance Delivery

Further methods have been developed to specifically target the area of endothelial inflammation or endothelial dysfunction. By covalently attaching receptors for markers expressed by endothelial cells during inflammation to the microbubbles, the bubbles attach and remain adjacent to the cells that require therapeutic cargo delivery. Activated endothelial cells have increased expression of ICAM-1, allowing it to be used as a binding target for microbubbles [139]. Conjugation of an ICAM-1 antibody to the microbubble surface resulted in significant binding to activated endothelial cells in culture under shear flow compared to no binding to healthy endothelial cells [139]. Conjugating VCAM-1 and E-selectin antibodies to microbubbles through a biotin-streptavidin reaction has also been evaluated for binding to activated endothelial cells [140]. Microbubbles conjugated to any of the three antibodies had significantly increased binding to human endothelial cells activated with 6 h or TNF-α treatment compared to untreated cells [140]. Microscopic imaging observed improved binding of antibody-conjugated microbubbles to activated human and mouse endothelial cells following exposure to the bubbles under flow conditions compared to non-conjugated microbubbles [140]. A potential method to improve the targeting of activated endothelial cells would be dual targeting with several of the previously mentioned antibodies. While Barriero et al. demonstrated dual targeting with endothelial markers (CD9 and ICAM-1), it has not yet been tested with both markers only present on activated endothelial cells and not healthy cells [141].

Targeted microbubbles have been especially useful in delivering therapeutic cargo to injured heart tissue. Using cationic microbubbles conjugated to P-selectin antibodies for targeting ischemic areas in a murine ischemia model and bound to a plasmid coding for hVEGF165, Shentu et al. delivered genetic cargo to the cardiac endothelium [142]. The study observed enhanced deposition of the genetic cargo resulting in improvement to cardiac function compared to without the targeting ligand [142]. A similar study successfully delivered the angiopoietin-1 gene using cationic microbubbles targeted with an ICAM-1 antibody to improve endothelial function and stimulate angiogenesis in an ischemic murine model [143]. 

### 2.2. A New Frontier? Ultrasound and Microbubble Treatment of the Injured Lung

**Figure 3:**
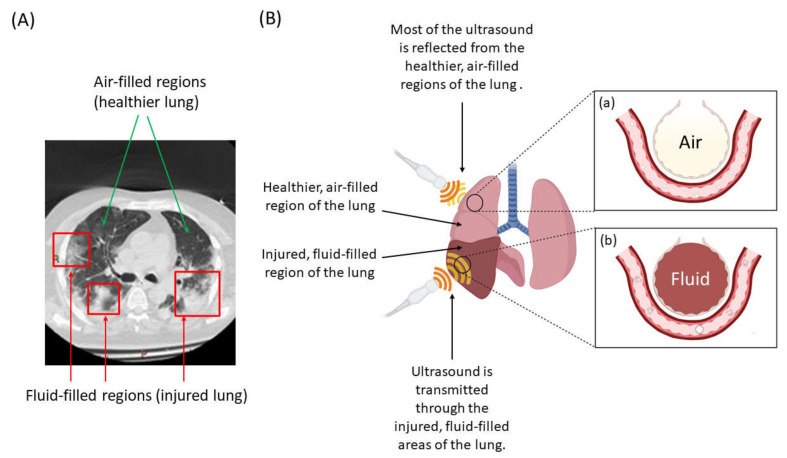
Courtesy of Laurent J. Brochard, St. Michael’s Hospital, Toronto(**A**) Lung CT scan (cross-sectional view) from a patient with ARDS showing heterogenous distribution of injured, fluid-filled regions of the lung (**B**) USMB treatment of the lung targets the most injured and edematous regions. (**a**) Ultrasound energy is unable to penetrate the relatively healthy aerated sections of the lung, reflecting instead. Microbubbles in this region flow through the capillaries unaffected by the ultrasound waves. (**b**) Ultrasound energy is able to penetrate when the alveoli are filled with fluid in edematous or de-aerated regions, sonicating the microbubbles. Cavitation of the microbubbles is induced, resulting in cellular uptake of therapeutic cargo preferentially in the most injured area of the lungs. Created with BioRender.com.

Despite the abundant literature describing ultrasound and microbubbles for cargo delivery across the blood–brain barrier or to tumors, there are very few reports of the technique in the lung. Early use of ultrasound in the lung focused on the study of pleural disease rather than lung tissue itself [144], since ultrasound is scattered by the air in healthy air-filled lung tissue. This has likely also delayed recognition of the potential of USMB to enhance drug delivery to the injured lung. In fact, lung injury or ARDS may be ideally suited to USMB-mediated therapy, since fluid leaking into the alveoli and the loss of air allows ultrasound waves to selectively penetrate the most injured lung regions [144]. Normal (aerated) or less-diseased regions of the lung would scatter the ultrasound energy, preventing or reducing off-target effects [145]. USMB is also likely to be effective for targeting densely consolidated lung (as in lobar pneumonia), even in the absence of diffuse disease (Figure 3).

The amount of penetration of ultrasound in the most injured, fluid-filled regions of the lung and normal, air-filled regions of the lung can be quantitatively estimated using acoustic impedance values of lung tissue, air and blood (Table 1) [146].

The amount of reflection and transmission of incident ultrasound wave at the lung–air interface and lung–fluid (i.e., blood) interface can be calculated using ultrasound reflection and transmission coefficients.

(1) $$R={\left(\frac{Z_{2}-Z_{1}}{Z_{2}+Z_{1}}\right)}^{2}$$

(2) $$T=\frac{4Z_{2}Z_{1}}{{\left(Z_{2}+Z_{1}\right)}^{2}}$$

where R is the ultrasound reflection coefficient and T is the ultrasound transmission coefficient. Here, Z_1_ and Z_2_ are acoustic impedance values of medium 1 and medium 2, respectively, at the interface that is exposed to the ultrasound wave. For the lung–air interface, Z_1_ can be assumed as the acoustic impedance of the lung while Z_2_ can be assumed as the acoustic impedance of the air. Similarly, for the lung–blood interface, Z_1_ can be considered as the acoustic impedance of the lung and Z_2_ can be considered as the acoustic impedance of the blood.

Using the above equations, it can be observed that almost the entire incident ultrasound wave (99.1%) is reflected back at the lung–air interface, while only 0.9% penetrates in the air-filled regions of the lung. On the contrary, 35.5% of the incident ultrasound penetrates the lung–blood interface, which is significantly higher than the penetration at the lung–air interface. These numbers quantitatively show that ultrasound energy penetrates the most injured areas of lungs, showing its potential for targeted drug and gene delivery. This remarkable selectivity of USMB for the most injured regions of the lung sets it apart from other potential endothelial-targeted therapeutic strategies such as nanoparticles or microparticles.

The first instance of USMB treatment for the delivery of therapeutic cargo to injured lung tissue was recently published by our group (2018), demonstrating delivery of the aminoglycoside antibiotic gentamycin in an E.coli-induced murine pneumonia model [147]. The study demonstrated an almost ten-fold reduction in bacterial colony-forming units following USMB treatment with gentamycin compared to gentamycin alone, an intriguing finding given that aminoglycoside antibiotics do not normally distribute well to the lung [147]. In fact, the dose of gentamicin that was administered was too low to inhibit bacterial growth in the absence of concomitant microbubbles and thoracic ultrasound [147]. USMB treatment significantly increased gentamicin concentrations in both bronchoalveolar lavage fluid and lung lysates [147]. In this study, USMB was also shown to have no detrimental effect on oxygenation or on the degree of lung injury scored in a blinded fashion by a lung pathologist [147]. These results of enhanced delivery to injured lung tissue were later validated in a rabbit model by a French group, who reported enhanced delivery of another aminoglycoside antibiotic, amikacin, to fluid-filled lung tissue [148]. Flooding the rabbits’ lungs with saline allowed the group to apply USMB treatment while administering amikacin at two doses, comparing delivery to sonicated and non-sonicated lung tissue [148]. USMB treatment significantly increased amikacin concentration in the sonicated lung tissue compared to the non-sonicated lung tissue, with a greater degree of enhancement observed at the lower amikacin dose [148]. Both of these studies highlight the enhancement of therapeutic cargo delivery by USMB at doses below the physiologically effective dosage.

The therapeutic benefits of delivering pulmonary surfactant (sinapultide) using USMB has also been investigated; delivery of sinapultide by insonation of sinapultide-loaded microbubbles reduced the severity of injury and levels of the inflammatory cytokines IL-6 and TNF-α in an LPS-induced model of lung injury [149]. Finally, USMB treatment has also been used to explore the therapeutic effects of delivering a VEGF antagonist (soluble fms-like tyrosine kinase-1) encapsulated in the microbubbles to a murine model of LPS-induced lung injury [150]. The lung injury group receiving USMB with soluble fms-like tyrosine kinase-1 exhibited improved P_a_O_2_, reduced lung injury score and wet-to-dry ratio, and a lower 7-day mortality rate compared to injured counterparts receiving USMB treatment with empty microbubbles [150]. This treatment was found to enhance endothelial barrier function by inhibiting the endothelial permeability caused by VEGF while avoiding off-target effects [150].

Despite these intriguing examples, however, the use of USMB in the injured lung still remains largely unexplored. Specifically, it remains to be seen whether other therapeutic cargoes such as plasmids and non-coding RNA can be harnessed with USMB to treat lung injury.

## 3. Future Directions—Challenges and Opportunities

### 3.1. Trade off of Increased Leakage vs. Cargo Delivery

One concern with USMB in the lung is the risk of increasing endothelial leakage or inflammation through sonoporation. Although sonoporation is transient, in theory it could aggravate tissue damage in the injured lung; on the other hand, sonoporation will also further enhance the delivery of therapeutic cargoes. Inflammation of the blood–brain barrier has been observed when treating with a microbubble dose 10 times higher than the clinical imaging dose; no damage was observed with the clinical dose [151]. The high microbubble dose also corresponded to increased contrast agent uptake in the surrounding tissue, indicating that increased inflammation and leakage allows for more cargo delivery [151]. Steroids such as dexamethasone have been investigated as a countermeasure to USMB-inflicted inflammation in the blood–brain barrier, with beneficial results [152]. Real-time microscopy of USMB-induced cultured endothelial barrier disruption observed the effect of shear stress on barrier permeability and how it can be manipulated through ultrasound frequency and microbubble oscillation dynamics [153]. Large microbubble-induced shear stress was determined to induce larger pores which could cause gap formation between cells [153]. Ultrasound settings, microbubble dosage and dynamics, and how often treatment is applied can all affect the degree of penetration and inflammation due to USMB treatment, as well as the amount of cargo delivery. While increasing the penetration has been linked to increased cargo delivery to underlying tissue, the safety of the procedure must be weighed against the effectiveness. Optimization of all of these parameters is recommended for the application of safe therapy while inducing cargo delivery with USMB.

### 3.2. Tissue (Depth) Penetration and Specificity for the Lung

Another feasibility issue is the degree to which thoracic ultrasound can penetrate the edematous or consolidated lung. Thoracic ultrasound has a maximum tissue penetration of about 10 cm, which would theoretically permit access to most of the average-sized human thorax (e.g., a chest circumference of 38 inches is a radius of ≈ 15 cm) [154]. In the event that surface ultrasound does not permit sufficient access, endobronchial ultrasound exists and would theoretically permit treatment of injured areas of the lung that are deep under the surface. For USMB to be used in clinical practice in humans, one could envisage development of a specific clam-shell-shaped ultrasound transducer that would permit simultaneous treatment of both the ventral and dorsal thorax. Finally, USMB is likely to be most effective in only the most severely injured lung regions; residual air in less severely injured areas of the lung is likely to block the ultrasound energy. Specifically, whether the technique will work in areas where interstitial (rather than alveolar) edema predominates is uncertain.

### 3.3. Optimizing Bubble Size and Charge for Delivery

Efficient delivery of cargo is affected by the properties of the microbubbles [155]. A particularly important variable is bubble surface charge. In lipid-shelled bubbles, the lipids chosen can impart a charge property on the bubble (e.g., 1,2-stearoyl-3-trimethylammonium-propane will lead to the generation of cationic microbubbles), and these properties have direct and measurable effects on the success of delivery. For example, genetic material in the systemic circulation is rapidly degraded, necessitating large amounts of genetic material to be injected, which may be unfeasible [156]. However, when nucleic acids were delivered with cationic microbubbles, their lifespan in the circulation was extended, leading to a lower required nucleic acid dose and a higher level of transfection [157,158,159,160,161,162]. This is a result of negatively charged nucleic acids coupling to the bubbles [163].

Bubble size is another important property that contributes to delivery capacity. In comparison to larger bubbles, smaller bubbles require higher ultrasound frequencies to undergo inertial cavitation [164]. Larger bubbles were found to achieve greater penetration depth of Evan’s blue dye and ascorbyl tetraisopalmitate in skin samples, suggesting that bigger bubbles are better for delivery [165]. However, large bubbles are more likely to cause detrimental blockage of the circulation; larger bubbles also result in more local tissue damage [166]. These consequences must be considered when choosing the right bubble size for a given application.

On the other end of the spectrum are nanobubbles, which are bubbles that are less than 1 micron in diameter [167]. Due to their very small size, they are able to traverse intercellular gaps and deposit deep into tissues, perhaps making them less desirable to endothelial transfection. They are desirable for applications such as tumor targeting, where they are able to infiltrate deep into the tumor for treatment [168]. They were also shown to be safer to cells than microbubbles, causing transfection in an in vitro model without impacting cell viability [169]. However, a major limitation of nanobubbles is the possibility of extravasation at other vascular sites, thus decreasing the amount deposited at the tissue of interest [170]. Nonetheless, their increased stability makes them an interesting avenue for endothelial delivery.

### 3.4. Emerging Techniques to Control Bubble Sizes and Charge

Most of the current USMB-mediated drug/gene delivery studies use commercial microbubble ultrasound contrast agents (such as SonoVue^®^, DEFINITY^®^, OPTISON^®^) [171]. Some studies also use custom-made bubbles which are prepared by either sonication or a mechanical agitation method [171]. This method of preparation often leads to polydisperse microbubbles with varying mean sizes [171]. The resonance frequency of microbubbles (i.e., ultrasound frequency at which the maximum amplitude of oscillation occurs) depends on the size of the microbubbles [86]. For example, smaller bubbles have a higher resonance frequency compared to larger bubbles, and the maximum amplitude of oscillation of smaller bubbles is less than larger bubbles because of increased damping [86]. Therefore, when polydisperse microbubbles are insonated, only a small fraction of the total population microbubbles resonate, which may negatively affect the drug/gene delivery efficacy [86]. One way to solve this problem is to use emerging microfluidic techniques which are capable of generating monodisperse microbubble populations. Using carefully designed microchannels and appropriate flow rates, monodisperse microbubbles of varying mean sizes can be generated at a high throughput [172,173,174]. Moreover, the shell properties (such as composition, charge) of the microbubbles can be varied by using different lipid mixtures in the continuous phase. Apart from microbubbles, microfluidics can also be used to generate monodisperse nanobubbles, as reported in a recent study [175]. Microfluidics thus provides a promising alternative to conventional agitation/sonication techniques for generating monodisperse bubbles, which could maximize the efficiency of USMB-mediated drug/gene delivery.

### 3.5. Clinical Trials of USMB Treatment for ARDS

The use of USMB treatment to enhance drug delivery for tissues in the digestive tract has been supported in recent clinical trials. Improvement in delivery of chemotherapeutic drug to target tissue has been demonstrated in pancreatic cancer, as well as various malignant tumors of hepatic and pancreatic organs [176,177]. These studies concluded that USMB-enhanced treatment improved patient outcome without additional toxicity or side effects compared to chemotherapeutic treatment alone. However, given the novelty of USMB treatment for injured lung tissue (first published in 2018), USMB has not yet been attempted in clinical trials for ARDS or lobar pneumonia [147]. Once sufficient in vitro and in vivo evidence has accumulated on the effectiveness and safety of USMB treatment for injured lung tissue, clinical trials are likely to follow [178].

## 4. Conclusions—A New Technique Provides New Opportunities

ARDS is a major cause of death after respiratory infection, whether from existing or emerging viral and bacterial pathogens. The pulmonary endothelium plays a significant role in the development and progression of ARDS, where loss of barrier integrity and excessive inflammation drive pulmonary edema. Current treatments are unable to specifically target the most injured pulmonary endothelium due to the heterogeneity of lung damage in any given patient. Because air scatters ultrasound energy, USMB-mediated cargo delivery is an attractive method that will preferentially target the most injured areas of the lung, sparing relatively aerated regions. Even in the absence of diffuse disease, USMB could also be used in the setting of a dense, lobar consolidation. In principle, this technique could be used to deliver small molecule or genetic agents that enhance endothelial barrier integrity, stimulate endothelial repair, and prevent excessive endothelial cell activation.

Finally, the ability to deliver drugs and potentially genetic material preferentially to non-aerated lung in vivo is likely to be useful in determining the pathogenesis of lung diseases even beyond ARDS. For instance, this technique might facilitate research into the pathogenesis and treatment of lung fibrosis or lung malignancy. Although the field is young, ultrasound-microbubble-mediated drug and gene delivery has the potential to be a valuable tool to understand and to treat the severely injured lung.

## 5. Patents

W.L.L. is listed as a co-inventor on a patent related to this work and is the Chief Scientific Officer of a spin-off company related to this field.

## Figures and Tables

**Table 1:** Acoustic impedance values of lung, air, and blood.

Medium	Acoustic Impedance
Lung	0.18 × 10^6^ Rayls
Air	0.0004 ×10^6^ Rayls
Blood	1.65 ×10^6^ Rayls

## Data Availability

No new data were created or analyzed in this study. Data sharing is not applicable to this article.
